# Characterization of a family mutation in the 5’ untranslated region of the endoglin gene causative of hereditary hemorrhagic telangiectasia

**DOI:** 10.1038/s10038-019-0564-x

**Published:** 2019-02-06

**Authors:** Lidia Ruiz-Llorente, Jamie McDonald, Whitney Wooderchak-Donahue, Eric Briggs, Mark Chesnutt, Pinar Bayrak-Toydemir, Carmelo Bernabeu

**Affiliations:** 1 Centro de Investigaciones Biológicas, Consejo Superior de Investigaciones Científicas (CSIC), and Centro de Investigación Biomédica en Red de Enfermedades Raras (CIBERER), 28040 Madrid, Spain; 2 ARUP Institute for Clinical and Experimental Pathology, and Department of Pathology, University of Utah, Salt Lake City, UT USA; 3 Departments of Medicine and Interventional Radiology, Oregon Health & Science University and Veterans Affairs Portland Health Care System, Portland, OR USA

**Keywords:** Disease genetics, Gene expression analysis

## Abstract

Hereditary hemorrhagic telangiectasia (HHT) is a vascular disease characterized by nose and gastrointestinal bleeding, telangiectases in skin and mucosa, and arteriovenous malformations in major internal organs. Most patients carry a mutation in the coding region of the endoglin (*ENG*) or activin A receptor type II-1 (*ACVRL1*) gene. Nonetheless, in around 15% of patients, sequencing analysis and duplication/deletion tests fail to pinpoint mutations in the coding regions of these genes. In these cases, it has been shown that sequencing of the 5’-untranslated region (5’UTR) of *ENG* may be useful to identify novel mutations in the *ENG* non-coding region. Here we report the genetic characterization and functional analysis of the heterozygous mutation c.-142A>T in the 5’UTR region of *ENG* found in a family with several members affected by HHT. This variant gives rise to a new initiation codon of the protein that involves the change in its open reading frame. Transfection studies in monkey cells using endoglin expression vectors demonstrated that c-142A>T mutation results in a clear reduction in the levels of the endoglin protein. These results support the inclusion of the 5’UTR of *ENG* in the standard genetic testing for HHT to increase its sensitivity.

## Introduction

Hereditary hemorrhagic telangiectasia (HHT) is an autosomal dominant vascular disease characterized by telangiectases, nose bleeds and arteriovenous malformations (AVMs). The presence of AVMs in lungs, liver, brain, or digestive tract can cause severe complications due to either blood shunting through AVMs or hemorrhage [1, 2]. Diagnosis of HHT can be made using clinical parameters. Thus, an individual is diagnosed with HHT when she/he has at least three out of four specific diagnostic criteria: (i) spontaneous-recurrent epistaxis; (ii) mucocutaneous telangiectases (especially on nose, tongue, lips, the mucous membrane lining the inside of the mouth, and fingers); (iii) AVMs in major internal organs (lung, liver, brain, gastrointestinal tract, spinal cord); and (iv) a first degree relative with established HHT diagnosis. When two of these criteria are present, the diagnosis is considered possible or suspected, whereas when there are fewer than two criteria, then HHT diagnosis is unlikely [1, 3]. In most cases, HHT is not life-threatening and symptoms can be effectively managed. However, HHT can be difficult to be diagnosed because it is clinically heterogeneous, with symptoms frequently differing even among family members [3].

HHT is a genetically heterogeneous disorder that affects different components of the TGF-β signaling pathway [4, 5]. Mutations in endoglin (*ENG*) and activin A receptor type II-like 1 (*ACVRL1* or *ALK1*), both transmembrane TGF-β receptors, cause HHT type 1 (HHT1; MIM 131195) and HHT type 2 (HHT2; MIM 601284), respectively [6, 7]. Together, *ENG* or *ACVRL1* account for the pathogenic mutations in over 80% of patients with HHT [5, 8–10]. HHT type 3 [11] and HHT type 4 [12] were designated based on linkage in one or two affected kindreds, with no gene identified to date. Also, mutations in the *SMAD4* gene, that encodes a transcription factor of the TGF-β-signaling pathway, are responsible for a combined syndrome of juvenile polyposis (JP) and HHT (JP-HHT; MIM600993), which occurs in less than 2% of HHT patients [13]. Moreover, mutations in the *GDF2* gene, that encodes the soluble protein BMP9, have been shown to cause an HHT-like phenotype (HHT5; MIM 615506) [14]. Interestingly, BMP9 is a member of the TGF-β family and can specifically bind to endoglin and ALK1 receptors. Overall, the HHT genetic subtypes share common symptoms; nonetheless, individual patients vary from each other in the first-time appearance, frequency, and severity of their vascular lesions, even within the same HHT subtype. HHT presents a high and age-dependent penetrance, so once an index case is diagnosed within the family, the molecular genetic analysis of *ENG*, *ACVRL1*, *SMAD4*, and *GDF2* can be used as a very predictive molecular diagnosis of the other at risk family members. Of note, some studies have found that a strategy of diagnostic genetic testing provides a more economically advantageous solution than the conventional clinical screening, because it reduces the number of clinical tests for those family members who do not have HHT [15].

Molecular diagnosis of HHT typically involves sequencing of *ENG* and *ACVRL1* coding regions, and large deletion/duplication analysis. Then, if no mutation is detected, analysis of *SMAD4* is carried out. However, more than 15% of HHT cases do not show mutations in coding regions of these genes [10]. Studies in some of these families have revealed the existence of mutations in non-coding DNA sequences such as introns or regulatory regions of *ENG* [http://arup.utah.edu/database/ENG/ENG_display.php; [16, 17]. In this sense, mutations in the 5’UTR of *ENG* [18] may account for the pathogenesis of HHT in some patients [19–21]. Supporting the critical regulatory role of this region is the fact that most of the protein complexes involved in transcription and/or translation bind and regulate expression from the 5’UTR of the gene. Here, we have identified a novel mutation in the 5’UTR region of *ENG* that generates a putative new translation initiation site, thus changing the open reading frame and interfering with the endoglin protein expression.

## Materials and methods

### Subjects

A single family with at least 5 affected individuals in 3 generations who met HHT clinical diagnostic criteria and were negative for an exonic mutation in *ENG*, *ACVRL1, SMAD4, GDF2/BMP9,* and *RASA1* was studied. Information regarding HHT symptoms and manifestations of family members was obtained during assessment at one or two HHT Centers of Excellence in North America (Yale University School of Medicine and Oregon Health & Science University). This study was approved by the Institutional Review Board of the University of Utah (IRB_00020480).

### Genomic DNA sequencing and plasmids

Genomic DNA was extracted via automated Magna Pure (Roche Diagnostics, Indianapolis, IN) from whole blood. Primers were designed to amplify the entire 5’UTR region of *ENG*, BigDye sequencing chemistry was used to sequence the PCR products in both directions using the ABI 3730xl DNA analyzer (Applied Biosystems, Foster City, CA). The sequences were analyzed by the Mutation Surveyor program (Softgenetics, State College, PA). The variants detected were compared to the NCBI dbSNP databases to determine if the nucleotide change found in our study had previously been reported in healthy individuals. The 3-kb fragment of human endoglin cDNA, containing the 5’UTR and cloned into *BbrPI*/*BamH1* sites of pCEXV vector, has been described [22]. This plasmid was used as a template to generate the single endoglin mutant c.142A>T using the following primers: 5′-AGATGGCTGGAGCAGGGACGC-3′ (Forward) and 5′-GCGTCCCTGCTCCAGCCATCT-3’ (Reverse). After site-directed mutagenesis, the parent template was removed using the methylation-dependent endonuclease *Dpn*I. The resulting mutant construct was confirmed by sequencing. The ENG mutant c.-127C>T in the expression vector pCEXV has been previously described [19].

### PCR and sequencing analysis of cDNA

Total RNA was extracted from peripheral blood mononuclear cells (PBMCs) and converted into cDNA using reverse transcriptase with random primers. Exonic primers (exons 1 and 2) were designed and used to specifically amplify and Sanger sequence the region of ENG that had the mutation. PCR amplification was carried out using High Fidelity PCR master mix (Roche) followed by automated electrophoresis analysis (QIAxcel Advanced, Qiagen). Primer sequences are shown in Supplementary Figure 1.

### Cell culture, transfections, and western blot analyses

The monkey kidney COS-7 cell line was cultured in DMEM supplemented with 10% heat-inactivated fetal calf serum, penicillin (100 U/mL) and streptomycin (100 µg/mL), in a humidified incubator with an atmosphere of 5% CO_2_–95% air at 37 °C. Cell transfections were carried out using Lipofectamine 2000 (ThermoFisher Scientific) as vehicle for plasmids, according to the manufacturer’s instructions. Forty eight hours after transfection, cells were lysed in lysis buffer and subjected to SDS-PAGE under reducing conditions, followed by immunoblotting with rabbit anti-endoglin (clone EPR10145-12, Abcam; 1:1000 dilution) or mouse anti-γ-tubulin (clone GTU-88, Sigma; 1:10,000 dilution) monoclonal antibodies in PBS-Tween with 5% BSA, as described [19]. The presence of the specific proteins was revealed with horseradish peroxidase conjugated anti-mouse or anti-rabbit IgG (Dako) and the reaction was developed by addition of SuperSignal chemiluminescent substrate (Thermo Scientific). Protein bands were visualized with a Chemi-Doc™ XRS+ equipment (Bio-Rad) and their intensity was quantified using Image Lab™ software. Results shown in the graphical representation are the mean of three different experiments. Data are expressed as mean ± SEM and p < 0.005 was considered to be statistically significant.

## Results

### Clinical and genetic studies of an HHT family

**Fig. 1:**
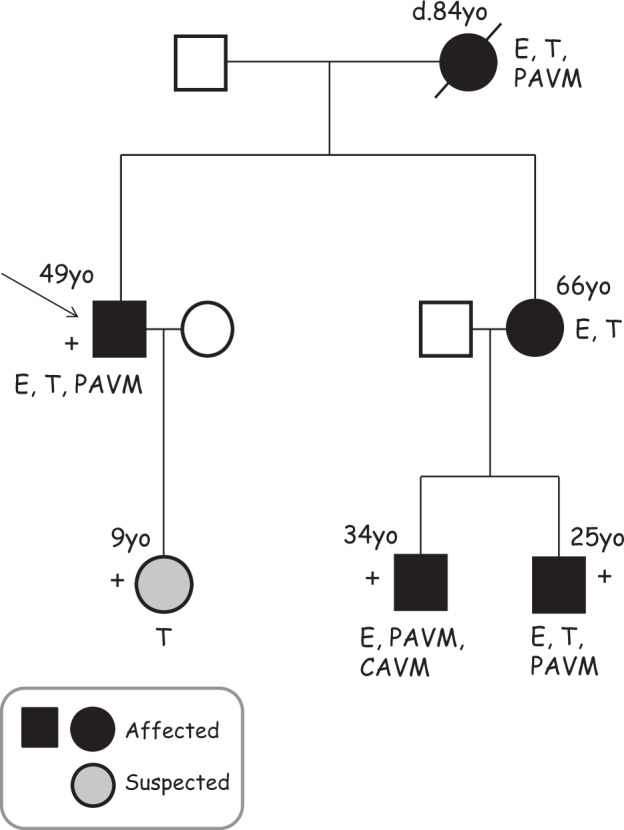
Family segregation study. The pedigree for the HHT1 family is shown. The c.-142A>T mutation (+) was shown to segregate among affected individuals in this family, where 5 clinically affected family members were available for this study. The age (years old, yo) of the individuals and their symptoms at the time of examination are indicated. CAVM, cerebral AVM; E, epistaxis; PAVM, pulmonary AVM, T, telangiectases

A family with at least 5 affected individuals was identified and evaluated at two different HHT Centers of Excellence. An HHT NGS gene panel (*ACVRL1*, *ENG*, *SMAD4*, *GDF2/BMP9*, and *RASA1*) failed to detect an exonic mutation. However, sequencing of the 5’UTR of *ENG* in the proband revealed the existence of the variant c.-142A>T (NM_000118.3(*ENG*):c.-142A> T) corresponding to the gDNA nomenclature Chr9(GRCh37):g.130616776T>A (Alamut® Visual; https://www.interactive-biosoftware.com/alamut-visual/). A search for the c.142A>T variant in the standard reference database for genetic variance GnomAD (http://gnomad.broadinstitute.org/) did not find this sequence change in the 5’UTR of *ENG*. Next, samples were collected from two affected nephews of the proband and the variant was shown to segregate with the disease in these family members (Fig. 1). Clinical manifestations of the proband and other assessed family members are also summarized in Fig. 1. Individuals included as having no solid organ involvement had previous screening for pulmonary AVMs using contrast echocardiography and/or chest computed tomography, and for brain AVMs by a contrast magnetic resonance imaging, as well as physical examination and medical history that did not suggest other AVMs.

**Fig. 2:**
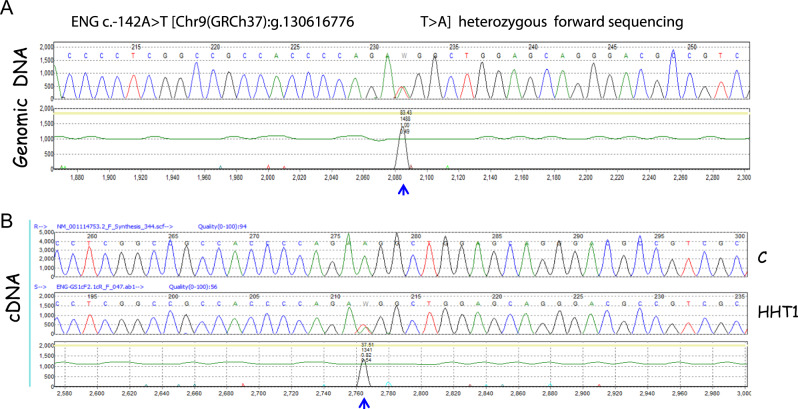
Genomic DNA and cDNA sequencing. **a** Genomic DNA sequencing results from one individual with the c.-142A>T heterozygous mutation. **b** Sequencing of cDNA isolated from peripheral blood leukocytes from an individual with the c.-142A>T heterozygous mutation (HHT1) or from a control subject (**c**). In both panels, the forward sequence is shown. The blue arrowhead indicates the position of the mutation

The schematic representation and neighboring genomic DNA sequences of the c.-142A>T variant are shown in Fig. 2a. The *ENG* mutation c.-142A>T was also detected at the mRNA level when sequencing cDNA derived from patient´s PBMCs (Fig. 2b). This Sanger sequencing analysis revealed almost identical intensity peaks of A and T nucleotides at the mutant position (blue arrowhead), suggesting similar levels of mRNA from both wild type and mutant alleles. This interpretation was supported by RT-PCR amplification of the cDNA using specific primers for the -142 nucleotide-containing region (encompassing from −401 to +219) involving *ENG* exon 1 and exon 2 (Supplementary Figure 1). As shown in Supplementary Figure 2, different combinations of primers always yielded a clear amplification of the mutant cDNA from the patient (red arrows), showing an intensity similar to the control sample. Taken together, these results suggest that the c.-142A>T mutation of *ENG* does not have a major impact on the transcription rate of the mutant allele.

**Fig. 3:**
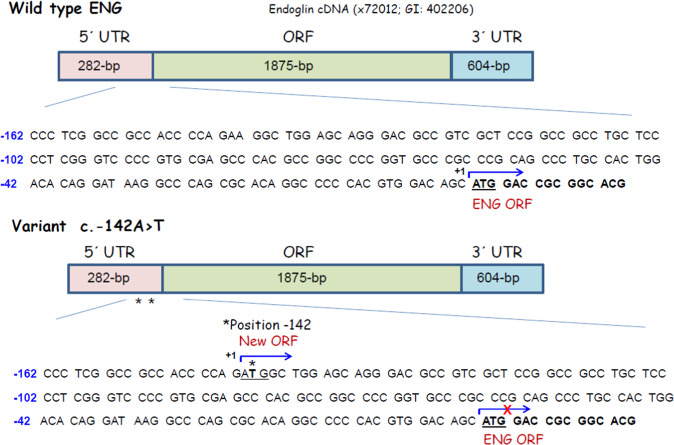
Schematic representation of endoglin mRNA. The 5’UTR, the 3’UTR and the open reading frame (ORF) are indicated in the diagram. Endoglin cDNA accession number (X72012) corresponding to the 3073-bp mRNA of endoglin [22] and the gene ID (GI, 402206) are included. The sequence of wild type (WT) and mutant c.-142A>T corresponding only to the −162/+15 region is shown. Asterisks indicate the positions of HHT1 mutant. The constitutive translation initiation site (ENG ORF) as well as the new putative translation initiation site of the mutant (+1; blue broken arrows) are underlined

**Fig. 4:**
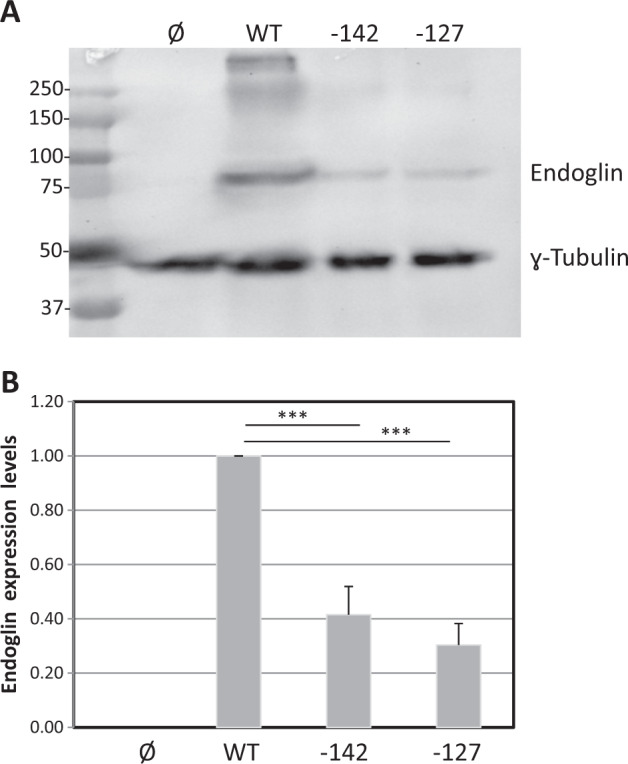
Functional analysis of the ATG endoglin mutant. COS-7 cells were transfected with wild type (WT) or endoglin mutants c-142A>T (−142) or c-127C>T (−127), using an empty vector (Ø) as a negative control. After 48 h, cells were lysed and total cell lysates were subjected to western blot analysis using anti-endoglin or anti-γ-tubulin antibodies. Normalized endoglin levels relative to total γ-tubulin protein from three different experiments are shown in the histogram. An arbitrary value of 1.0 was assigned to WT endoglin. ***p < 0.005

Next, we investigated the possible effect of this mutation on the protein translation process. This sequence change creates a putative AUG initiation codon at base −141 from the constitutive translation initiation of the *ENG* gene. The c.-142A>T change is not reported in the NCBI dbSNP database. Interestingly, the NetStart 1.0 Prediction Program (http://www.cbs.dtu.dk/services/NetStart/) [23] predicts that this mutation creates a new functional translation start site (TIS) with an altered reading frame (Fig. 3). Of note, the flanking sequence of the new TIS fits well with the Kozak consensus sequence and other motifs that contribute to the translation initiation process [23, 24], suggesting that this new TIS may be functional. As translation preferentially initiates at the first ATG codon in an appropriate context, it is likely that the TIS at −141/−143 is competing advantageously with the constitutive TIS at +1. In order to test this hypothesis, we generated a mutant construct in a full-length endoglin cDNA, that contains the 5’UTR [22], where the c.-142A>T change was introduced, as described in Materials and methods. The expression vectors encoding the wild type and mutant cDNA were transiently transfected in the monkey cell line COS-7, and the protein expression levels of endoglin were assessed. As depicted in Fig. 4, the protein levels of the mutant endoglin construct c.-142A>T were markedly reduced (60%) with respect to the wild type construct. As expected, an expression vector encoding the *ENG* mutation c.-127C>T, known to generate a new and pathogenic TIS [19], also yielded a markedly reduced expression of endoglin in the same experiment. These results suggest that the c.-142A>T mutation generates an early functional TIS out of frame that prevents translation initiation of the constitutive ATG at +1, resulting in endoglin haploinsufficiency.

## Discussion

Molecular diagnosis of HHT is currently based on the sequencing of coding regions of the three known causative genes [1, 4, 10]. However, ~15% of clinically diagnosed HHT patients do not show mutations in the coding regions or exon/intron junctions of *ACVRL1*, *ENG*, or *SMAD4*, in spite of the fact that in some families, linkage analyses suggest *ACVRL1* or *ENG* to be the causative gene [10, 25]. These findings clearly suggest that non-coding regions may also play a role in the disease. In fact, several reports have described the existence of HHT causing mutations in the 5’UTR and introns of *ENG* [http://arup.utah.edu/database/ENG/ENG_display.php; [16, 17, 19–21]. Among these are variants that involve the 400-bp upstream from the TIS of *ENG*, a region near the transcription initiation site and essential for *ENG* promoter function [18]. By sequencing this region, we have identified a 5’UTR mutation (c.-142A>T) in 4 different members of the same HHT1 family showing co-segregation with symptoms. Affected individuals in this family had classical clinical criteria of HHT disease, including involvement of internal organs.

It is commonly accepted that most disease-causing mutations in *ENG* lead to haploinsufficiency [1, 4, 5]. Accordingly, HHT1 likely results from the lack of sufficient endoglin protein, not reaching the threshold for normal function. Thus, mutations resulting in mRNA degradation or in structural alterations associated with misfolding and/or intracellular degradation of the protein, lead to an impaired cell surface expression of the functional endoglin. The c.-142A>T mutation in the 5’UTR creates a new TIS resulting in an out-of-frame product. Accordingly, translation initiation from this novel start site predicts a new protein with no homology to the wild type protein and with a premature stop codon. The consequence of this mutation would be similar to that of any of the frameshift mutations seen in *ENG*, which result in lack of endoglin protein expression on the cell surface. Indeed, expression studies confirmed that endoglin protein levels were decreased to 40% of the wild-type control values. This finding is in agreement with quantitative measurements of endoglin protein levels in cultured endothelial cells derived from HHT1 patients [4].

Kozak sequences are conserved motifs recognized by ribosomes as the start of protein translation [23, 24]. Because the constitutive TIS of *ENG* does not show a robust Kozak consensus sequence and protein translation usually initiates at the first ATG codon, this suggests that the new TIS at −141 is competing advantageously with the constitutive TIS at +1.

5’UTR mutations that alter the initiation codon have been reported as pathogenic mutations for other genetic disorders [26–28]. In HHT1, three mutations (c.-9G>A, c.1-10C>T and c.-127C>T), likely involving the generation of a new initiation codon upstream of the constitutive TIS, have been published [19, 20, 29], whereas no similar mutations have been described in the 5’ UTR of *ACVRL1*, the gene mutated in HHT2. Of note, many mutations leading to truncation and frameshift may result in nonsense-mediated decay and therefore reduced mRNA levels [30]. Thus, in addition to the effects on endoglin protein translation/processing, the possibility that these ATG mutations may also decrease mRNA stability cannot be ruled out, as described in HHT1 for several truncation mutations of *ENG* [31]. In addition, we cannot exclude a putative effect of the c.-142A>T mutation in the transcriptional regulation of ENG. To address this, we compared wild type and mutated sequences with in silico prediction tools, aiming at identifying changes in putative transcription factor binding sites centered in the −142 nucleotide. We found the −142A>T change generates a putative consensus binding site for HOXA3 (CCAGATGGC) with 100% score and 0 mismatches (Patch1.0). Interestingly, HOXA3 regulates gene expression and function in embryonic and adult endothelial cells [32, 33], which are the target cells in HHT [1, 4, 5]. Whether this HOXA3 sequence of ENG is transcriptionally active *in vivo* remains to be analyzed.

In summary, we have identified a novel pathogenic mutation in the 5’UTR region of *ENG*, which adds further evidence to previous studies supporting the inclusion of this region in standard genetic testing for HHT to improve clinical sensitivity.

## Supplementary information

Supplementary Information
