# Supplementary material for: Characterization of a family mutation in the 5’ untranslated region of the endoglin gene causative of hereditary hemorrhagic telangiectasia
Source: J Hum Genet. 2019 Feb 6;64(4):333–9. doi: 10.1038/s10038-019-0564-x (PMC8075931; doi:10.1038/s10038-019-0564-x)
Supplement: Supplementary file 1 — Supplementary Information [file 10038_2019_564_MOESM1_ESM.pdf]

A

ENG cDNA: **NM_001114753**

GGCAGGCGGCCTGGCCCAGCCCCTTCTCTAAGGAAGCGCATTTCCTGCCTCCCTGGGCCG *ex01cF*

GCCGGGCTGGATGAGCCAGGAGCTCCCTGCTGCCGGTCATACCACAGCCTTCATCTGCGC

CCTGGGGCCAGGACTGCTGCTGTCACTGCCATCCATTGGAGCCCAGCACCCCCTCCCCGC

CCATCCTTCGGACAGCAACTCCAGCCCAGCCCCGCGTCCCTGTGTCCACTTCTCCTGACC

CCTCGGCCGCCACCCCAGAAGGCTGGAGCAGGGACGCCGTCGCTCCGGCCGCCTGCTCCC *ex01cF_wt/mut*

CTCGGGTCCCCGTGCGAGCCCACGCCGGCCCCGGTGCCCGCCCGCAGCCCTGCCACTGGA

CACAGGATAAGGCCCAGCGCACAGGCCCCCACGTGGACAGCATGGACCGCGGCACGCTCC

CTCTGGCTGTTGCCCTGCTGCTGGCCAGCTGCAGCCTCAGCCCCACAA

GTCTTGCAGAAACAGTCCATTGTGACCTTCAGCCTGTGGGCCCCGAGAGGGGCGAGGTGA *ex02cR*

CATATACCACTAGCCAGGTCTCGAAGGGCTGCGTGGCTCAGGCCCCCAATGCCATCCTTG *ex02.1cR*

AAGTCCATGTCCTCTTCCTGGAGTTCCCAACG

B

Primer name	Sequence (5’ to 3’)
ENG_ex01cF	CTTCTCTAAGGAAGCGCATTT
ENG_ex01cF_wt	TCGGCCGCCACCCCAGAA
ENG_ex01cF_mut	TCGGCCGCCACCCCAGAT
ENG_ex02cR	GCTGAAGGTCACAATGGACT
ENG_ex02.1cR	GACATGGACTTCAAGGATGG

Supplementary Figure 1. Primers used to amplify the ENG cDNA sequence. **A.** The cDNA sequence subject to study, corresponding to exon 1 (upper text; -401 to +67) and exon 2 (lower text; +68 to +219) is shown. The nucleotide position at -142 of the A>T mutation is underlined. Exon 1 forward (F) primers are marked with blue, while exon 2 reverse (R) primers are marked with yellow. **B.** The sequences of the primers and their names are indicated.

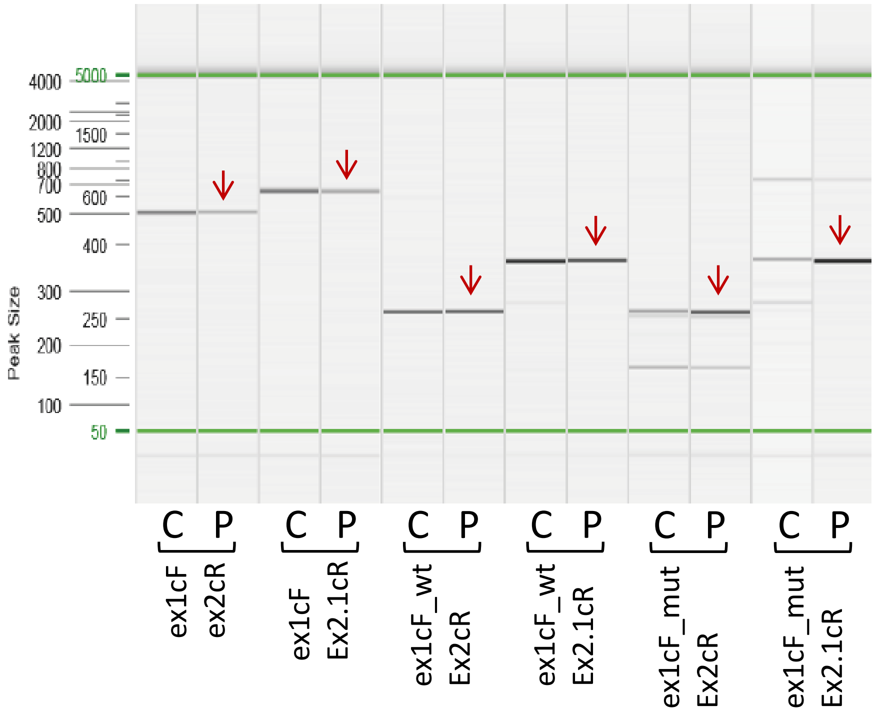

Supplementary Figure 2. PCR analysis of ENG cDNA. Samples of total RNA isolated from PBMCs of patient (P) and control (C) subjects were subjected to RT-PCR. Amplification was performed using different combinations of forward and reverse primers located in ENG exon 1 and exon 2, respectively (Supplementary Figure 1), as indicated. Clear amplification products, containing the nucleotide at -142, were observed with patient (red arrows) and control samples. The DNA ladders indicate the size of the DNA markers.
